# Vicarious Menstruation

**Published:** 1850-10

**Authors:** 


*Vicarious Menstruation.*—In the June number of the Western Lancet, Dr. B. F. Richardson, of Cincinnati, reports a case in which at each regular menstrual period the discharge takes place from the anus instead of the vagina, and continues about as long, and in quantity is about the same as the normal menstrual discharge. The fluid does not coagulate. Its discharge is usually preceded by pain and uneasiness in the lower part of the bowels. From careful and minute inquiry, Dr. R. is satisfied that the discharge was not hemorrhoidal, and that there was no reason to suspect a recto-vaginal communication. A very singular case of vicarious menstruation was brought to the notice of the Westminster Medical Society by Dr. Rogers, last year, in which blood oozed from the tips of the fingers. —*Southern Med. and Surg. Journal.*

